# Synbiotic *Bacillus megaterium* DSM 32963 and n-3 PUFA Salt Composition Elevates Pro-Resolving Lipid Mediator Levels in Healthy Subjects: A Randomized Controlled Study

**DOI:** 10.3390/nu16091354

**Published:** 2024-04-30

**Authors:** Bodo Speckmann, Tanja Wagner, Paul M. Jordan, Oliver Werz, Manfred Wilhelm, Heike tom Dieck, Christiane Schön

**Affiliations:** 1 Evonik Operations GmbH, Rodenbacher Chaussee 4, 63457 Hanau, Germany; 2 BioTeSys GmbH, Schelztorstrasse 54-56, 73728 Esslingen, Germany; 3 Department of Pharmaceutical/Medicinal Chemistry, Institute of Pharmacy, Friedrich Schiller University Jena, 07743 Jena, Germany; 4 Jena Center for Soft Matter (JCSM), Friedrich Schiller University Jena, 07743 Jena, Germany; 5 Department of Mathematics, Natural and Economic Sciences, Ulm University of Applied Sciences, 89081 Ulm, Germany

**Keywords:** omega-3, probiotic, metabiotic, *Bacillus megaterium*, inflammation resolution, specialized pro-resolving mediators, nutritional therapy

## Abstract

Beneficial health effects of omega-3 polyunsaturated fatty acids (*n*-3 PUFA) are partly attributed to specialized pro-resolving mediators (SPMs), which promote inflammation resolution. Strategies to improve *n*-3 PUFA conversion to SPMs may, therefore, be useful to treat or prevent chronic inflammatory disorders. Here, we explored a synbiotic strategy to increase circulating SPM precursor levels. Healthy participants (n = 72) received either SynΩ3 (250 mg eicosapentaenoic acid (EPA) plus docosahexaenoic acid (DHA) lysine salts; two billion CFU Bacillus megaterium; n = 23), placebo (n = 24), or fish oil (300 mg EPA plus DHA; N = 25) capsules daily for 28 days in a randomized, double-blind placebo-controlled parallel 3-group design. Biomarkers were assessed at baseline and after 2 and 28 days of intervention. The primary analysis involved the comparison between SynΩ3 and placebo. In addition, SynΩ3 was compared to fish oil. The synbiotic SynΩ3 comprising Bacillus megaterium DSM 32963 and *n*-3 PUFA salts significantly increased circulating SPM precursor levels, including 18-hydroxy-eicosapentaenoic acid (18-HEPE) plus 5-HEPE, which was not achieved to this extent by fish oil with a similar *n*-3 PUFA content. Omega-3 indices were increased slightly by both SynΩ3 and fish oil. These findings suggest reconsidering conventional *n*-3 PUFA supplementation and testing the effectiveness of SynΩ3 particularly in conditions related to inflammation.

## 1. Introduction

Omega-3 polyunsaturated fatty acids (*n*-3 PUFA), including primarily alpha-linoleic acid (ALA), eicosapentaenoic acid (EPA), and docosahexaenoic acid (DHA), are ingested through the regular diet (e.g., in seafood, flaxseed, canola, and nuts) and through widespread application of dietary supplements. Numerous intervention trials have been performed with *n*-3 PUFA supplements, showing potential health benefits, including reducing the risk of cardiovascular outcomes, improving cognitive function, and reducing inflammation [1,2]. The recommended daily intake of *n*-3 PUFA varies depending on age, sex, and health status. It ranges from 200 mg to 4 g, according to different healthcare authorities such as the American Heart Association [3]. Several studies showed a large variation in response towards *n*-3 PUFA supplementation [4,5,6], arguing for more advanced or personalized, instead of generic supplementation strategies. This response variability can be attributed to, e.g., differences in baseline intake, duration of supplementation, dosage, form, and formulation of the *n*-3 PUFA preparations, and intrinsic factors like gender, health status, and age.

Furthermore, *n*-3 PUFA after intake is further metabolized by several endogenous enzymes to bioactive lipid mediators (LMs) [7]. A prominent group of LMs are the so-called specialized pro-resolving mediators (SPMs), which are di- or trihydroxylated products of EPA and DHA. The SPM family can be divided into resolvins (RV), protectins, and maresins as well as arachidonic acid (AA)-derived lipoxins, whose biosynthesis involves lipoxygenases, cytochrome P 450 monooxygenases, and cyclooxygenase-2 (see [8,9,10] for an overview of the pathways). SPMs promote the resolution of inflammation by various receptors and mechanisms [8]. Additional functions have been ascertained to select monohydroxylated SPM precursors like 5-hydroxy-eicosapentaenoic acid (HEPE) and 18-HEPE, such as the inhibition of monocyte adhesion to vascular endothelial cells [11], inhibition of pressure-overload-induced maladaptive cardiac remodeling [12], and induction of adipose tissue browning [13]. SPMs can, therefore, be considered important mediators of the health-beneficial effects of *n*-3 PUFA, particularly concerning the amelioration or prevention of cardiovascular and chronic inflammatory conditions as well as pain perception [14]. Importantly, the body’s capacity to synthesize or respond to SPM can be compromised, as shown for, e.g., inflammatory bowel diseases [15], metabolic syndrome [16,17], arthritis [18], and polycystic ovary syndrome [19] and this may contribute to persistent inflammation. On the other hand, the physiological capacity to produce SPM precursors like 18-HEPE may determine the clinical response towards *n*-3 PUFA, as shown for EPA in major depressive disorder [7].

Strategies aimed at increasing the concentration of SPMs in the body could represent a promising nutritional or therapeutic approach. However, previous *n*-3 PUFA studies have shown significant increases in SPMs (in plasma or serum) only at supraphysiological *n*-3 PUFA doses [20,21,22] or after a lipopolysaccharide endotoxin challenge [23]. The same applies to the direct administration of exogenous SPMs in the form of SPM-enriched fish oil preparations, which showed only a short-term increase in plasma SPM concentrations at very high doses [24]. 

As an alternative, we recently developed a synbiotic strategy based on a microbial conversion of *n*-3 PUFA to SPMs by probiotic bacteria of the genus *Bacillus megaterium*, which acts self-sufficiently and independent of host metabolism [25]. An important aspect of this strategy is to enable a long-lasting and sustained supply of endogenously formed SPMs, which is advantageous over short-term peak concentrations using classical SPM-enriched fish oil preparations. In a pilot study, a significant increase in plasma concentrations of several SPM precursors, including 18-HEPE and 5-HEPE, was observed after low-dose administration of a formulation comprising the SynΩ3 combination [25]. Here, we investigated the effect of SynΩ3 in a controlled study compared to a fish oil preparation with a comparable *n*-3 PUFA content, as well as to a placebo. We hypothesized that supplementation with SynΩ3 leads to significantly higher plasma levels of 18-HEPE and 5-HEPE as compared to fish oil or placebo.

## 2. Materials and Methods

### 2.1. Study Design

The study was conducted as a double-blind, randomized, placebo-controlled parallel design with a 4-week intervention period. Study visits were performed at baseline, after 2 days and at the end of the 4-week intervention. The study participants were either allocated to the double-blind groups of the study receiving placebo or verum or to the exploratory additional comparator study arm with commercial fish oil (ratio 1:1:1). The randomization list was generated with RandList (DatInf GmbH, Tübingen, Germany) stratified for gender with a blocksize of six. According to the randomization list, the pill boxes were labelled with the subject number. Subject numbers were consecutively allocated to subjects according to their inclusion. The study was performed at the study site of the Nutritional CRO BioTeSys GmbH (Esslingen, Germany). All subjects gave their informed consent before they participated in the study. The study was approved by the Institutional Review Board (IRB) of Landesärztekammer Baden-Württemberg (Reference number: F-2022-078) and conducted in accordance with the guidelines for Good Clinical Practice (GCP) set forth by the International Council for Harmonisation of Technical Requirements for Pharmaceuticals for Human Use (ICH), and in accordance with the Declaration of Helsinki regarding the treatment of human subjects in a study. Registration: German register DRKS00030106.

### 2.2. Study Subjects

**Figure 1:**
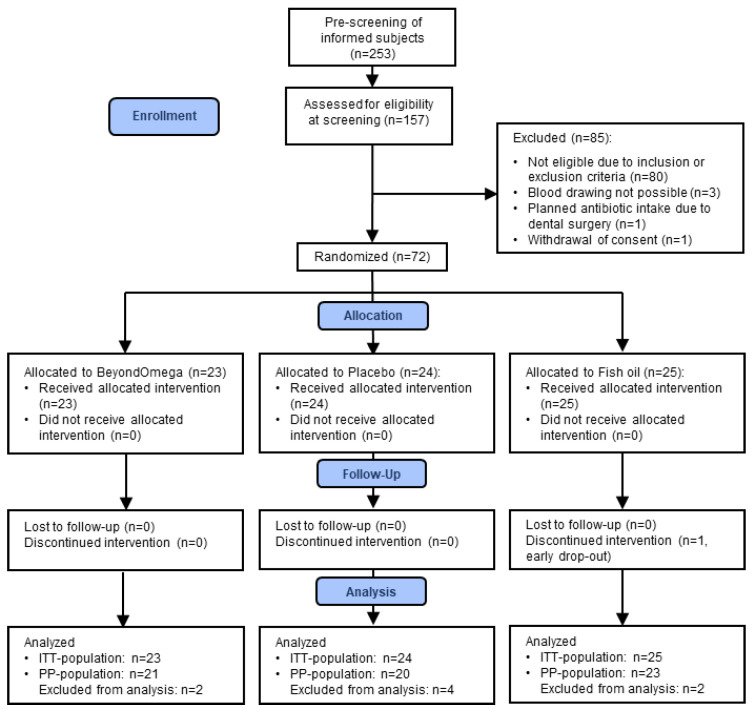
Study flow chart.

Between September 2022 and March 2023, 157 subjects were screened for their eligibility. Out of these, 85 were screening failures, and 72 (healthy males and females) were included in the study. Therefore, 72 subjects were considered for the intention-to-treat (ITT) population and 64 subjects for the per-protocol (PP) population (Figure 1). Data are presented for the ITT population. The main inclusion criteria were age between 30 and 75 years, a BMI of 19–35 kg/m^2^, and classification as an at-risk individual defined as at least slightly elevated advanced glycation end products (AGEs) and/or high sensitive C-reactive protein (hsCRP) level ≥ 1 mg/L. AGEs and hsCRP are commonly used biomarkers to assess oxidative stress and inflammation [26,27], which determine the risk and progression of various diseases, such as diabetes. Only nonsmokers were allowed to participate. As the plasma levels of several pro-resolving LMs usually correlate with the omega-3 index [28], only subjects with a cut-off level of omega-3 index ≤ 6.5% were enrolled in the study. To control for confounding factors, subjects were asked to refrain from fish consumption or other *n*-3 PUFA supplements or probiotics during the study intervention and also to avoid plant oils rich in *n*-3 PUFA. Furthermore, they were asked to maintain their food and lifestyle habits. Blood routine parameters were determined at the screening visit. Only volunteers with no clinically relevant abnormal findings and blood values within defined ranges were enrolled in the study. The clinical relevance of blood routine parameters outside the reference range was judged by the Investigator. The main exclusion criteria were as follows:

(1) Relevant history or presence of any severe medical disorder, potentially interfering with this study (e.g., malabsorption, chronic gastro-intestinal diseases, heavy depression, diabetes, acute cancers within the last 3 years except basal cell carcinoma of the skin, etc.);
(2) Subject under prescription for medication or taking dietary supplements possibly interfering with this study (such as *n*-3 PUFA, probiotics, anti-spasmodic, laxatives and anti-diarrheic drugs or other digestive auxiliaries, use of PPIs, bismuth salts and/or H2-antagonists, fibers, chronic intake of NSAR or acetylsalicylic acid, glucocorticoids, etc.) within 2 weeks before study start or during the study;
(3) Intake of antibiotics in the last 2 months and laxatives in the last 2 weeks.

### 2.3. Study Products

Investigational product: The investigational product synbiotic omega-3 (SynΩ3) is a dietary supplement in capsule form (IN VIVO BIOTICS™ resolvin Beyond Omega). One capsule contained 1 billion colony-forming units of *Bacillus megaterium* DSM 32963 (B4U™63), *n*-3 PUFA lysine salt containing 83.3 mg EPA, 41.7 mg DHA (AvailOm^®^), and 27.5 µg selenium in the form of L-selenomethionine. The capsules were coated for targeted delivery of the ingredients into the intestine (EUDRAGUARD^®^ biotic functional coating for colon delivery). 

Placebo: Cellulose powder was used as a placebo, provided in titanium-dioxide-free vegetarian HPMC capsules. The capsules were coated for targeted delivery of the ingredients into the intestine (EUDRAGUARD^®^ biotic functional coating for colon delivery). The investigational product (SynΩ3) and placebo product were blinded. SynΩ3 and the placebo were manufactured by Evonik Operations GmbH, Hanau, Germany. 

Reference product fish oil: The fish oil product “Seefischöl Omega-3500 mg” by Zein Pharma was used at a dosing of 2 capsules per day equivalent to a daily intake of 180 mg EPA and 120 mg DHA in triglyceride form and 2.7 mg alpha-tocopherol (Vitamin E). As generic fish oil capsules on the market were used, this arm was not blinded to the investigational and placebo arm. Nevertheless, analogous to verum and placebo, the products were dispensed in neutral flasks so that the study site staff did not know which subject was receiving which study product when dispensing the study products.

For each type of study product, one capsule was taken in the morning (flexible with or without breakfast) and one capsule in the evening (flexible with or without dinner) unchewed with water. 

On the study day, prior to study visits 2 and 3, the evening capsule was standardized and taken 12 ± 2 h before the scheduled visit at the study site. 

The subjects were asked to document the consumption of the study products daily in a diary. Furthermore, residual study products were returned and counted. Compliance was calculated based on the returned products and for SynΩ3 per detection of *Bacillus megaterium* DSM 32963 in feces.

### 2.4. Methods for Samples and Data Collection

#### Blood Sampling

Blood sampling was performed at each visit after at least 10 h of overnight fasting for the analysis of biomarkers and blood routine parameters.

### 2.5. Analysis of Lipid Mediators, Omega-3 Index, and n-3 PUFA

#### 2.5.1. Analysis of Lipid Mediators in Plasma

Lipid mediator (LM) analysis using ultra-performance liquid chromatography-tandem mass spectrometry (UPLC-MS/MS) was performed as described previously [29] with some minor modifications. Briefly, freshly thawed human plasma or *Bacillus megaterium* cultures were first mixed with the same volume of ice-cold methanol containing deuterium-labeled internal standards (200 nM d8–5S-HETE, d4-LTB_4_, d5-LXA_4_, d5-RvD2, d4-PGE_2_ and 10 µM d8-AA; Cayman Chemical/Biomol GmbH, Hamburg, Germany) to facilitate quantification and sample recovery. Samples were kept at −20 °C for 60 min to allow protein precipitation. After centrifugation (1200× *g*, 4 °C, 10 min), 8 mL of acidified water was added (final pH = 3.5) and the samples were subjected to solid-phase extraction (SPE). The SPE cartridges (Sep-Pak^®^ Vac 6cc 500 mg/6 mL C18; Waters, Milford, MA, USA) were equilibrated with 6 mL of methanol and then 2 mL of water before sample loading onto the columns. After washing with 6 mL of water and an additional 6 mL of *n*-hexane, LMs were eluted with 6 mL of methyl formate. The eluates were brought to dryness using a TurboVap LV evaporation system (Biotage, Uppsala, Sweden) and resuspended in 100 µL of methanol/water (50/50, *v*/*v*) for analysis using UPLC-MS-MS. The LMs were analyzed with an Acquity™ UPLC system (Waters, Milford, MA, USA) and a QTRAP 5500 Mass Spectrometer (ABSciex, Darmstadt, Germany), equipped with a Turbo V™ Source and electrospray ionization. LMs were separated using an ACQUITY UPLC^®^ BEH C18 column (1.7 µm, 2.1 × 100 mm; Waters, Eschborn, Germany) at 50 °C with a flow rate of 0.3 mL/min and a mobile phase consisting of methanol/water/acetic acid of 42/58/0.01 (*v*/*v*/*v*) that was ramped to 86/14/0.01 (*v*/*v*/*v*) over 12.5 min and then to 98/2/0.01 (*v*/*v*/*v*) for 3 min. The QTrap 5500 was operated in negative ionization mode using scheduled multiple reaction monitoring (MRM) coupled with information-dependent acquisition. The scheduled MRM window was 60 sec, optimized LM parameters were adopted, and the curtain gas pressure was set to 35 psi. The retention time and at least six diagnostic ions for each LM were confirmed using external standards (Cayman Chemical/Biomol GmbH, Hamburg, Germany). Quantification was achieved with calibration curves for each LM. Linear calibration curves were obtained for each LM and gave r^2^ values of 0.998 or higher (for PUFA 0.95 or higher). Additionally, the limit of detection for each targeted LM was determined [30]. Parameters with concentrations below the limit of detection were considered with a limit of detection for further statistical analysis. 

#### 2.5.2. Omega-3 Index and Total *n*-3 PUFA in Plasma

The omega-3 index is the sum of EPA and DHA expressed as a percentage of the total fatty acids in erythrocyte membranes. In recent years, the omega-3 index has been recognized as a new biomarker for quantifying long-term *n*-3 PUFA intake and correlates very well with *n*-3 PUFA content in other tissues. At screening, analysis of the omega-3 index in erythrocytes was performed using gas chromatography at Omegametrix GmbH to select subjects. 

During the intervention, analysis of the omega-3 index in erythrocytes and plasma fatty acid analysis were performed at OmegaQuant LLC (Sioux Falls, SD, USA) using gas chromatography (GC) with flame ionization detection. Plasma fatty acid analysis was performed as previously described [31]. The fatty acid composition was expressed as a percentage of the total identified fatty acids and concentration (µg/mL) in the plasma. The omega-3 index was measured using the HS-Omega-3 Index^®^ methodology as previously described [32].

### 2.6. Inflammatory Biomarkers in Blood

HsCRP was determined using a turbidimetric method in serum at the routine lab (Synlab Leinfelden, Leinfelden-Echterdingen, Germany). Tumor necrosis factor (TNF)-alpha was measured in serum samples using a chemoluminescent immunoassay at Synevo Studien Service Labor GmbH, Berlin, Germany.

### 2.7. Statistical Analysis 

Based on a previous pilot study [25], the effect size for the increase in the two main SPM precursors 18-HEPE and 5-HEPE was estimated with d = 0.96. Applying a two-tailed approach and power of 80%, a sample size of 24 subjects was estimated considering a dropout rate of 20% for the primary comparison of investigational product versus placebo. This sample size was also applied for the exploratory comparison of SynΩ3 with a conventional fish oil product. 

ANCOVA models were applied for the changes between the end of intervention and baseline accounting baseline values as a covariate. In the case of non-normality of residuals, the Wilcoxon rank sum test was applied. The changes after two days of intervention were investigated with the same approach. Furthermore, the differences between placebo and SynΩ3 were also investigated for 18-HEPE and 5-HEPE with a linear mixed model with repeated measures for changes over time and baseline conditions considered as covariate. In a backward elimination, the impact of further covariates like gender, age, and BMI were evaluated. 

For the exploratory comparison of SynΩ3 with fish oil, an unpaired *t*-test or, if applicable, non-parametric Wilcoxon rank sum test was applied. In addition, changes over time within groups were investigated with repeated measurement ANOVA or the Friedman test in case of three assessments during the study conduct or paired-test or corresponding non-parametric approach if only baseline and end-of-intervention measures were performed. In the figures, the statistical significance from an investigation of within-group changes and pairwise tests against baseline are shown with * *p* < 0.05, ** *p* < 0.01, and *** *p* < 0.001.

## 3. Results

### 3.1. Study Collective

A total of 72 healthy subjects (25 men and 47 women) were enrolled between October 2022 and May 2023. There was one drop-out in the course of the study; the other 71 subjects all completed the study according to the protocol. Characteristics of study participants are summarized in Table 1. 

There were no differences in age or BMI between groups. On average, the omega-3 index was 4.80% in the placebo group, 5.07% in the SynΩ3 group, and 4.83% in the fish oil group. 

The overall compliance in the study was very good. Only in the SynΩ3 group, one subject showed a compliance of <80%. The compliance in the SynΩ3 group was also indirectly confirmed by the determination of *Bacillus megaterium* DSM 32963 in stool samples. Thus, in 21 of 23 subjects, the probiotic could be determined using qPCR. Medication, lifestyle, dietary habits, and metabolic parameters were similar between all three groups and did not significantly change during the study.

### 3.2. Primary Efficacy Endpoint: Significant Increase in the Sum of 18-HEPE and 5-HEPE by SynΩ3

**Figure 2:**
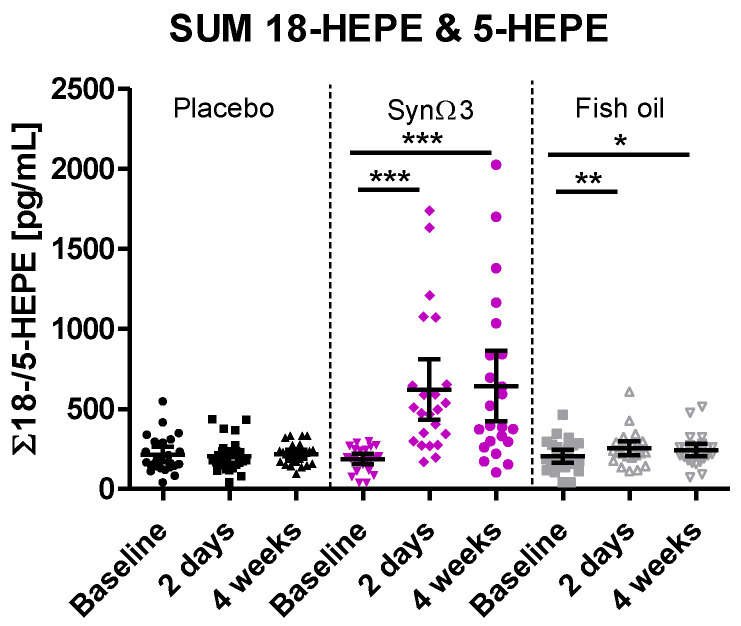
Plasma levels of the sum of 18-HEPE and 5-HEPE at baseline, after 2 days, and after 4 weeks of intervention; scatter diagram with mean ± 95% CI; * *p* < 0.05, ** *p* < 0.01, *** *p* < 0.001; significance derived from investigation of within-group changes and pairwise tests against baseline.

The EPA-derived 18-HEPE and 5-HEPE are the monohydroxylated precursors of RvE1 and RvE2, 18-HEPE is the precursor of RvE3, and 5-HEPE is the precursor of RvE4. The SPM precursors 18-HEPE and 5-HEPE were found to be the most abundant LMs generated from EPA. At baseline, low concentrations of 18-HEPE and 5-HEPE could be determined in the plasma of most of subjects. Only in some individual subjects were concentrations below the limit of detection. During the intervention with the placebo, the sum of 18-HEPE and 5-HEPE plasma concentrations (defined as the primary endpoint) remained unchanged over time (Figure 2). However, during supplementation with SynΩ3, a highly significant increase in 18-HEPE/5-HEPE levels was seen by an average factor of 3.3 after 2 days of supplementation, which was confirmed by a factor of 3.5 after 4 weeks of supplementation; see Table 2. Despite the supplementation of comparable amounts of *n*-3 PUFA provided by the fish oil, only a minor increase in 18-HEPE and 5-HEPE levels by an average factor of 1.2 after 2 days and 4 weeks of supplementation was observed (Figure 2 and Table 2). The increase in plasma levels of 18-HEPE plus 5-HEPE was much higher after intake of SynΩ3 in comparison to placebo and fish oil; see Figure 2 and Table 2.

### 3.3. Treatment Effects on Selected Lipid Mediators

**Figure 3:**
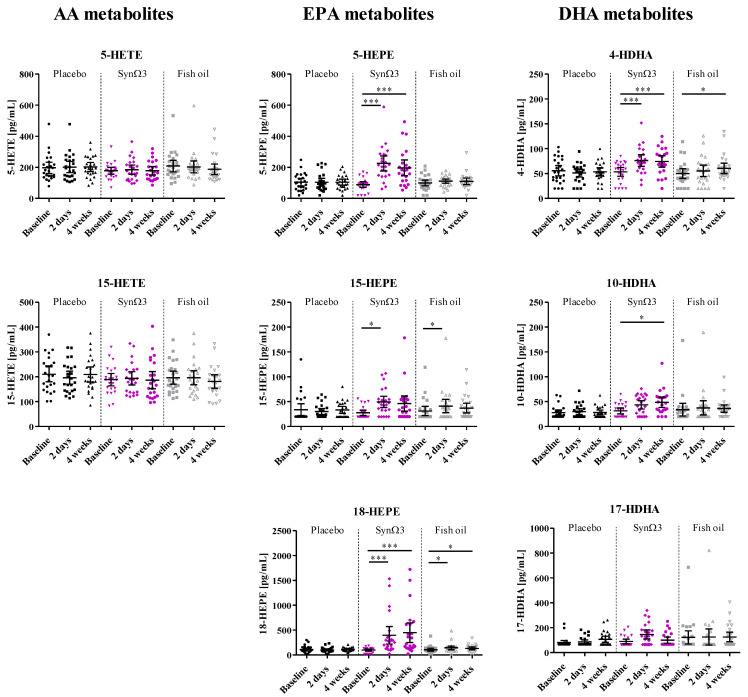
Plasma levels of individual lipid mediators at baseline, after 2 days, and after 4 weeks of intervention; scatter diagram with mean ± 95% CI; *** *p* < 0.001; * *p* < 0.05; significance derived from the investigation of within-group changes and pairwise tests against baseline.

Next, we assessed the plasma levels of individual LMs, including 18-HEPE, 5-HEPE, and other SPM precursors as well as relevant metabolites derived from EPA, DHA, and AA; see Figure 3 and Table 2.

The concentrations of 18-HEPE and of 5-HEPE at baseline were comparable. Of note, after supplementation with either SynΩ3 or fish oil, the formation of 18-HEPE dominated in comparison to 5-HEPE. This is in line with our previous study [25] and with the in vitro characterization of a *Bacillus megaterium*-derived CYP enzyme as a producer of 18-HEPE from EPA [33]. For all EPA-derived metabolites (5-HEPE, 15-HEPE, and 18-HEPE), a highly significant increase was seen after supplementation with SynΩ3 (18-HEPE: factor 4.1 (after 2 days), factor 4.6 (after 4 weeks); 5-HEPE: (factor 2.5 (after 2 days), factor 2.2 (after 4 weeks)) in contrast to a minor increase in fish oil (18-HEPE: factor 1.4 (after 2 days), factor 1.3 (after 4 weeks); 5-HEPE: factor 1.1 after 2 days and 4 weeks) (Figure 3 and Table 2). For the primary comparison of SynΩ3 against placebo, the significant differences in the levels of 18-HEPE and 5-HEPE between the groups were confirmed with a linear mixed model with repeated measures. Further impacting factors like gender, age, and BMI were investigated. For 18-HEPE, the covariate age impacted by trend (*p* = 0.0713); for 5-HEPE, the covariate age was significant (*p* = 0.0061). In contrast, gender and BMI did not show a significant impact.

In analogy to EPA-derived metabolites, a significant increase was also observed for the DHA-derived LMs, i.e., 4-HDHA and 10-HDHA after supplementation with SynΩ3. For fish oil, only a significant increase in 4-HDHA was observed. For other DHA-derived LMs, the levels were rather low in the majority of subjects with no significant changes between treatments. For the AA-derived metabolites, no changes over time were seen in any of the study groups, confirming the selective modulation of EPA- and DHA-derived metabolites during supplementation with SynΩ3 or fish oil. For all investigated LMs produced from EPA and DHA, higher levels after supplementation of SynΩ3 in comparison to fish oil were evident. Superiority could be demonstrated for the SPM precursors 18-HEPE and 5-HEPE after two days and four weeks of intervention. For the HDHA metabolites 4-HDHA, 10-HDHA, and 17-HDHA, superiority was demonstrated after two days of supplementation, but differences were minor pronounced between products after 4 weeks of intervention; see Table 2.

### 3.4. Modulation of n-3 PUFA Levels in Blood Compartments

To determine whether the observed differential modulation of LMs could be explained by differences in substrate availability among the treatment arms, we assessed EPA and DHA levels in three different blood compartments: (i) as a percentage of the erythrocyte membrane total fatty acid content (omega-3 index); (ii) as total plasma EPA and DHA, esterified and free fatty acids (FFA); (iii) in the form of the FFA in plasma only.

**Figure 4:**
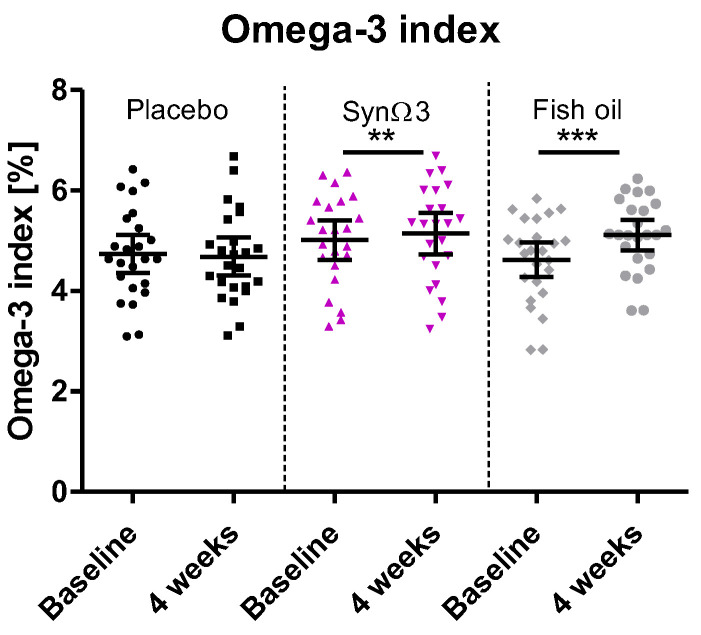
Distribution of omega-3 index (%) at baseline and after 4 weeks of intervention; scatter diagram with mean ± 95% CI; ** *p* < 0.01, *** *p* < 0.001; significance derived from investigation of within-group changes.

The omega-3 index was significantly increased by 4-week supplementation of SynΩ3 or fish oil (Figure 4). Changes after supplementation with SynΩ3 were significant in comparison to the placebo (*p* = 0.0088), whereas supplementing with fish oil was superior to SynΩ3 in terms of the elevation of the omega-3 index (SynΩ3: change over time: 0.12%; fish oil: change over time: 0.5% (*p* < 0.0001)).

**Figure 5:**
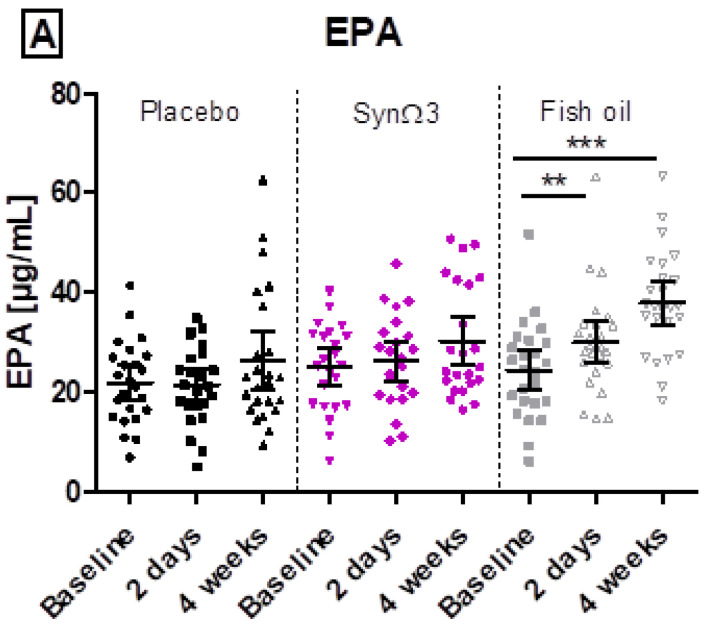

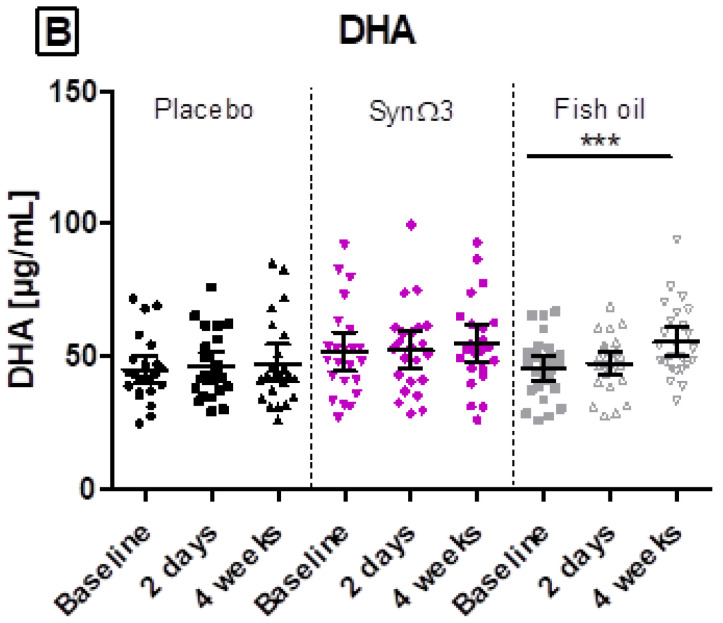
Plasma levels of (**A**) total EPA and (**B**) total DHA (μg/mL) at baseline, after 2 days and 4 weeks of intervention; scatter diagram with mean ± 95% CI; ** *p* < 0.01, *** *p* < 0.001; significance derived from the investigation of within-group changes and pairwise tests against baseline.

**Figure 6:**
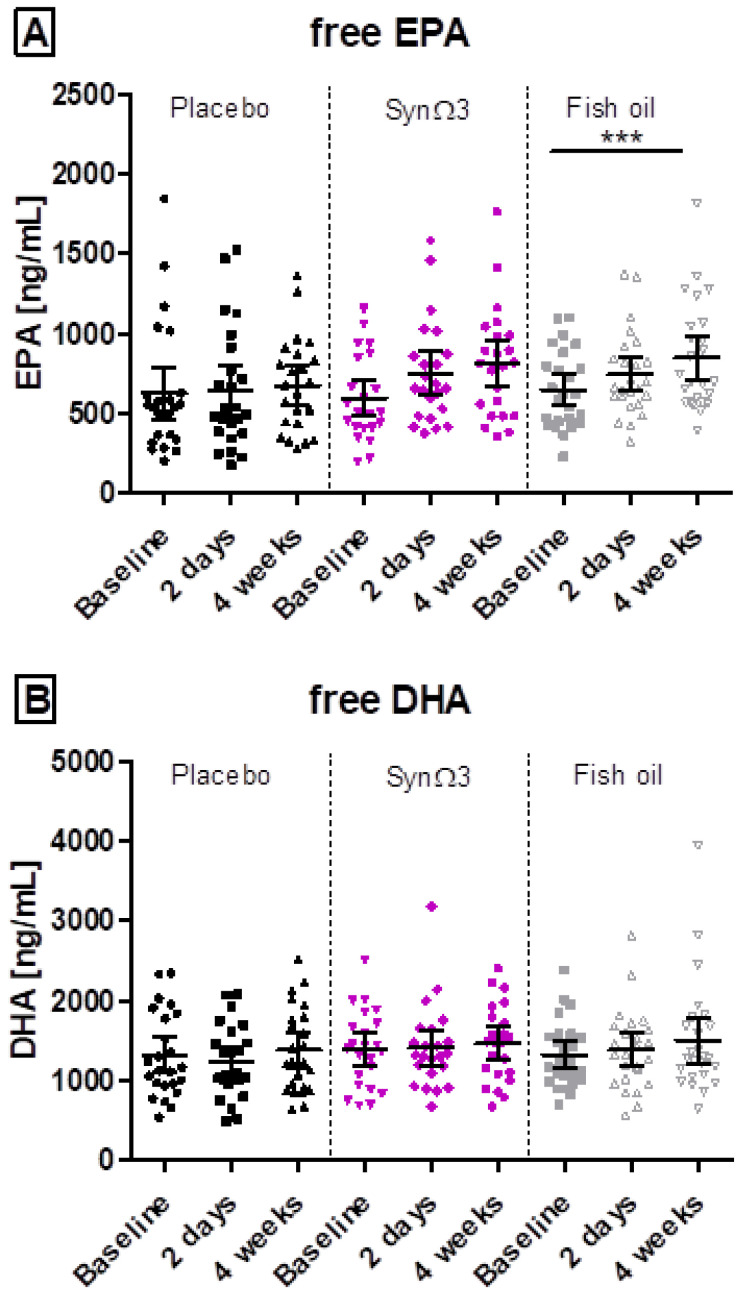
Plasma levels of (**A**) free EPA and (**B**) free DHA (ng/mL) at baseline, after 2 days and 4 weeks of intervention; scatter diagram with mean ± 95% CI; *** *p* < 0.001; significance derived from the investigation of within-group changes and pairwise tests against baseline.

During supplementation with SynΩ3, only a minor increase in total EPA and DHA plasma levels was observed, which was neither significant over time (Figure 5 and Figure 6) nor in comparison to placebo after 4 weeks of supplementation (Supplementary Table S1 and Figure 5 and Figure 6). In contrast, a highly significant increase in total EPA and DHA levels was observed for fish oil. Between SynΩ3 and fish oil, a significant difference in total EPA levels was documented after 2 days (−4.5 μg/mL difference in mean change SynΩ3 versus fish oil; *p*-value < 0.0001) and 4 weeks (−8.4 μg/mL difference in mean change SynΩ3 versus fish oil; *p*-value = 0.0055) of supplementation. For total DHA levels, only after 4 weeks of supplementation a significant difference (−6.8 μg/mL; *p*-value = 0.0173) was seen for the mean changes between groups (Supplementary Table S1).

### 3.5. EPA and DHA as Free Fatty Acids in Plasma

Additionally, the levels of EPA and DHA as FFA in plasma were measured; see Figure 6. In contrast to the total EPA levels, differences by trend could be observed for the delta change in EPA FFA after supplementation of SynΩ3 in comparison to the placebo, which was confirmed after 2 days and 4 weeks of supplementation.

Comparing the changes between SynΩ3 and fish oil, a comparable increase in EPA and DHA FFA was observed with no significant differences between products for EPA and DHA after 2 days and 4 weeks of supplementation. Note that the free forms of EPA and DHA are the substrates for the biosynthesis of LMs, including SPMs and their precursors.

### 3.6. Evaluation of Cholesterols and Triglycerides

The lipid/cholesterol status was determined at baseline and end of intervention. Baseline levels of total- and LDL-cholesterol varied between groups, but within all groups, these levels did not change significantly. In the placebo group, a statistically significant increase in HDL concentration was evident (*p* = 0.0175), which also contributed to the significant group difference after four weeks of intervention between the placebo and the SynΩ3 group (Table 3). In the SynΩ3 and fish oil groups, no differences over time were observed.

In contrast, triglycerides decreased upon supplementation with *n*-3 PUFA, both in the SynΩ3 and fish oil group (−12 mg/dL). Statistical significance over time was only reached in the SynΩ3 group (*p* = 0.0216). In the placebo group, an increase in triglycerides was observed (Table 3).

### 3.7. Evaluation of TNF-α and hsCRP

**Figure 7:**
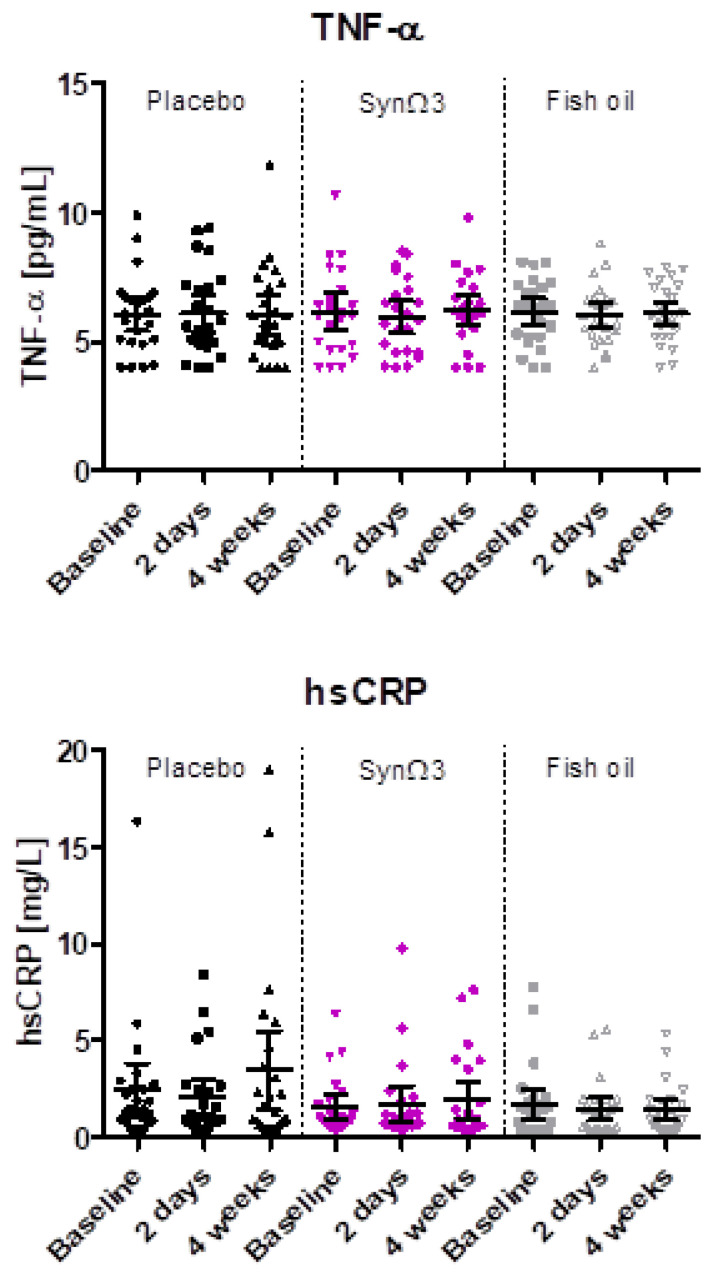
Plasma levels of TNF-α (pg/mL) and hsCRP (mg/L) at baseline, after 2 days, and after 4 weeks of intervention; scatter diagram with mean ± 95% CI.

As at-risk individuals were enrolled in the study, defined as at least slightly elevated AGEs and/or a hsCRP level ≥ 1 mg/L, we tested whether the study products display possible effects on the pro-inflammatory markers hsCRP and TNF-α. Overall, no significant changes over time in hsCRP and TNF-α plasma concentrations were observed in any of the study groups (Figure 7). The observed difference by trend (*p*-value: 0.0862) between mean changes in hsCRP in SynΩ3 and fish oil after 4 weeks of intervention is mainly due to the slight increase in the SynΩ3 group; in contrast, in the fish oil group, average concentrations slightly decreased (Supplementary Table S2).

### 3.8. Safety and Tolerability

To monitor safety concerns, tolerability and adverse events were inquired about at study visits. Tolerability was assessed at the end of the intervention phase. Overall, the tolerability of all study products was very good. There was only one subject per group who complained about slightly unpleasant or very unpleasant tolerability; reasons for these judgments included the following: heartburn after intake (placebo group); a feeling of pressure, burping, and/or flatulence (SynΩ3 group); and a fishy aftertaste (fish oil group).

During the study, a total of 87 adverse events (AEs) were reported by 42 subjects. One of these AEs was classified as a serious adverse event (SAE) due to hospitalization. The most AEs in the intervention phase were reported in the placebo group (n = 36), followed by the fish oil group (n = 33) and the SynΩ3 group with only n = 18 AEs. In the total study collective, the most commonly reported AE was headache/migraine (n = 38). One subject (SynΩ3 group) reported burping, flatulence, and a feeling of pressure in the stomach during the intervention phase, which was rated as possibly related to the intake of the study product. All other AEs were not related to the intake of the investigational products. Altogether, only one AE (elevated HbA1c in the diabetic range) conflicted with study participation, and this subject was excluded from the study at visit 2 (drop-out). No other AEs were in conflict with study participation and the study was continued according to the protocol.

## 4. Discussion

*n*-3 PUFA intake—either via the diet or through the use of supplements/functional food products—has long been practiced for preventing or ameliorating conditions arising from chronic or excessive inflammation, such as metabolic syndrome (MetS) and cardiovascular diseases. However, recent meta-analyses of clinical data have challenged the view that EPA/DHA supplementation has beneficial effects in humans. As a consequence, the design of current *n*-3 PUFA studies has become more specific, e.g., by selecting certain subject collectives, special formulations, and taking confounding factors more into account. In this context, we reasoned that the conversion of *n*-3 PUFA to SPMs and their precursors such as 18-HEPE and their subsequent signal transduction events are crucial determinants of *n*-3 PUFA outcomes. The literature describes numerous examples of disturbed levels, production capacity, and activity of SPMs in the diseased versus healthy state (summarized in [25,34,35]). Furthermore, the clinical response to *n*-3 PUFA is stronger in patients with a greater ability to produce LMs, as shown for 18-HEPE in major depressive disorder [7], which again suggests addressing the conversion of *n*-3 PUFA.

We previously introduced a novel route towards endogenous *n*-3 PUFA conversion, based on a recently described concept of synbiotics that exploits specific metabolic properties of probiotic strains [36]. More precisely, our route applies the CYP102A1-dependent metabolic capacity of *Bacillus megaterium* to produce a range of SPMs and precursors from exogenously provided *n*3-PUFA in a strain-specific manner. Thus, a formulation comprising a synbiotic combination of *Bacillus megaterium* DSM 32963 and *n*-3 PUFA lysine salt increased plasma concentrations of, e.g., 5-HEPE, 15-HEPE, and 18-HEPE, in a pilot study with healthy adults [25]. Our current study aimed at confirming this finding in a controlled study and comparing the quantitative effect of SynΩ3 against a fish oil supplement with a similar *n*-3 PUFA content. Directly comparing the pro-resolving LM production efficacy of conventional and synbiotic *n*-3 PUFA supplementation was vital because plasma concentration measurements of SPMs and their precursors greatly rely on the choice of the analytical platform [37].

18-HEPE and 5-HEPE, in sum and individually, were significantly increased in plasma by an average factor of 3.3–3.5 after two days and four weeks of supplementation with SynΩ3. The stronger increase in 18-HEPE levels (4.6-fold after four weeks) compared to 5-HEPE is in agreement with our pilot study as well as the fact that the CYP enzyme contained in *Bacillus megaterium* has been described to convert EPA to 18-HEPE in vitro before [33]. In our study, despite comparable amounts of EPA provided by the fish oil, only a minor increase in 18-HEPE and 5-HEPE by an average factor of 1.2 after two days and after four weeks of supplementation was observed. The latter finding is in line with previous human studies, wherein significant increases in 18-HEPE and other LMs were only found at very high doses (1.0–4.4 g per day) of esterified *n*-3 PUFA, SPM-enriched *n*-3 PUFA, isolated EPA, or in conjunction with an endotoxin challenge [7,20,21,22,23,24,28,38]. Interestingly, the EPA-derived 15-HEPE, the precursor of RvE4, and the DHA-derived 4-HDHA and 10-HDHA were also elevated in plasma from subjects supplemented with SynΩ3, but not or much less in the placebo or fish oil group. This supports the requirement of *Bacillus megaterium* for elevating these monohydroxylated EPA- and DHA-derived LMs on top of the supplementation of EPA/DHA. In fact, the corresponding monohydroxylated AA-metabolites 5-HETE and 15-HETE were not increased in the plasma of the SynΩ3 group, implying a certain selectivity for conversion of the *n*-3-PUFA EPA and DHA (but not of the *n*-6-PUFA AA) by *Bacillus megaterium*. Despite the increase in pro-resolving LMs, we did not observe a change in inflammatory biomarkers in the blood, which is due to the rather healthy population having relatively low baseline concentrations of, e.g., TNF-α and hsCRP.

Strikingly, in the OMEGA-PAD I Trial, daily ingestion of 4.4 g (2.6 g EPA and 1.8 g DHA) *n*-3 PUFA nearly doubled the omega-3 index but increased plasma levels of 5-HEPE, 15-HEPE, and 18-HEPE by only 1.9-fold, 1.7-fold, and 2.3-fold, respectively [28]. This underlines that maximizing the intake or bioavailability of *n*-3 PUFA is not the most expedient strategy for robustly increasing the concentration of (circulating) pro-resolving LMs. In this context, the greater formation of 18-HEPE from SynΩ3 versus fish oil observed in our study cannot be explained by higher bioavailability of EPA, as the increase in free plasma EPA was comparable, and the increases in the total EPA and omega-3 index were even significantly higher after fish oil intervention. We therefore reason that gut luminal production of these LMs in the SynΩ3 group, seemingly due to *Bacillus megaterium*, explains this difference. Though we have not confirmed this hypothesis experimentally in vivo, our previous studies clearly show the capability of *Bacillus megaterium* strains to produce SPMs and the precursors 18-HEPE and 5-HEPE in vitro [25]. It would be worthwhile to follow up on this by assessing the SPM profile in other relevant body compartments/fluids (e.g., intestinal mucosa, serum, urine, feces, etc.) in subsequent human trials or advanced gut models. In particular, and due to the increase in 18-HEPE and 5-HEPE, the formation of the related SPM RvE1, RvE2, and RvE4 needs to be studied in this respect.

## 5. Conclusions

In summary, we have shown the superiority of a targeted synbiotic approach to increase the plasma concentration of circulating pro-resolving LMs in middle-aged, healthy but at-risk subjects. This effect did not occur with a standard fish oil with a similar *n*-3 PUFA content and without added *B. megaterium*. Our findings strengthen the idea that a disturbed or limited metabolic capacity of the body to produce pro-resolving LM with health-beneficial properties from *n*-3 PUFA can be compensated by suitable microbes or synbiotics. The clinical implication of this is that the outcomes of *n*-3 PUFA supplementation, especially in the prevention and treatment of chronic inflammatory conditions, may be significantly improved through this synbiotic approach. Additionally, targeted metabolism allows for much smaller doses of *n*-3 PUFA than typically applied, which avoids possible side effects from supranutritional intake levels and increases the convenience and therefore patient compliance. Testing the impact of this synbiotic formulation on other body compartments and patients will provide additional and crucial insights into its pharmacokinetic and pharmacodynamic SPM profile and possible therapeutic use, e.g., for cardiovascular diseases, rheumatoid arthritis, and inflammatory bowel diseases.

## 6. Patent Applications

WO2020109474A1

WO2020109480A1

## Figures and Tables

**Table 1:** Study participant characteristics.

Variables	Study Population Mean (± 95% CI)
Age (years)	54.5 (51.9–57.1)
BMI (kg/m^2^)	25.7 (24.8–26.6)
Cholesterol (mg/dL)	219.7 (209.7–229.6)
LDL-cholesterol (mg/dL)	146.8 (136.5–157.1)
GPT (U/L)	26.7 (24.3–29.1)
GOT (U/L)	26.1 (24.7–27.5)
hsCRP (mg/L)	1.9 (1.4–2.4)
Systolic blood pressure (mmHg)	126.2 (122.5–129.9)
Diastolic blood pressure (mmHg)	79.2 (76.8–81.6)
Omega-3 index in erythrocytes (%)	4.9 (4.7–5.1)
AGE in skin	31.9% no risk 55.6% risk group 1 5.6% risk group 2 6.9% risk group 3

BMI: body mass index; LDL: low-density lipoprotein; GPT: glutamate pyruvic transaminase; GOT: glutamic oxaloacetic transaminase; AGE: advanced glycation end products, CI: confidence interval.

**Table 2:** Statistical analysis of individual lipid mediators in plasma at baseline, after 2 days, and after 4 weeks of supplementation presented by study groups. Mean ± 95% CI. Differences in mean changes between SynΩ3 and placebo as well as SynΩ3 and fish oil are shown.

Lipid Metabolites		Assessment Points	Placebo (n = 24) Mean (95% CI)		SynΩ3 (n = 23) Mean (95% CI)		Fish Oil (n = 25) Mean (95% CI)		Difference in Mean Change SynΩ3 vs. Placebo	*p*-Value ^§^	Difference in Mean Change SynΩ3 vs. Fish Oil	*p*-Value ^#^
**AA metabolites**	**5-HETE** **[pg/mL]**	**Baseline**	**196.8**	(160.9, 232.6)	**178.4**	(156.4, 200.5)	**208.4**	(171.9, 244.9)				
		2 days	**201.8**	(166., 237.5)	**184.8**	(154.5, 215.2)	**203.3**	(164.8, 241.9)	1.4	**0.3580**	11.5	**0.4712**
		4 weeks	**202.9**	(173.8, 231.9)	**178.2**	(150.7, 205.6)	**187.5**	(153.4, 221.7)	−6.3	**0.8670**	20.7	**0.2357**
	15-HETE ** **[pg/mL]**	Baseline	**210.2**	(180.3, 240.)	**188.4**	(163.7, 213.2)	**196.5**	(170.1, 222.9)				
		2 days	**196.6**	(170.6, 222.5)	**193.9**	(167., 220.8)	**196.5**	(168.3, 224.8)	19.1	**0.3949**	5.5	0.3491 *
		4 weeks	**209.6**	(179.7, 239.5)	**186.5**	(151.9, 221.1)	**181.4**	(154.8, 208.0)	−1.3	0.5728 *	13.2	**0.4186**
**EPA metabolites**	**18-HEPE+5-HEPE** **[pg/mL]**	Baseline	**214.0**	(164.9, 263.1)	**185.8**	(152.4, 219.3)	**204.8**	(164.5, 245.0)				
		2 days	**204.3**	(160.0, 248.7)	**620.7**	(432.7, 808.8)	**254.1**	(211.4, 296.8)	444.6	<0.0001 *	385.6	<0.0001 *
		4 weeks	**216.4**	(187.6, 245.1)	**642.5**	(420.9, 864.1)	**242.4**	(202.2, 282.7)	454.3	<0.0001 *	419.1	0.0003 *
	**18-HEPE** **[pg/mL]**	Baseline	**109.0**	(78.1, 139.8)	**97.0**	(75.4, 118.6)	**105.1**	(75.8, 134.5)				
		2 days	**99.8**	(77.5, 122.1)	**395.2**	(218.5, 571.9)	**142.3**	(105.5, 179.1)	307.4	<0.0001 *	261.0	0.0003 *
		4 weeks	**112.0**	(96.3, 127.6)	**446.2**	(247.7, 644.6)	**132.2**	(105.2, 159.2)	346.2	0.0007 *	322.1	0.0032 *
	**5-HEPE** **[pg/mL]**	Baseline	**105.0**	(81.6, 128.5)	**88.8**	(70.5, 107.1)	**99.6**	(80.7, 118.5)				
		2 days	**104.5**	(79.5, 129.6)	**225.5**	(177.3, 273.8)	**111.8**	(97.6, 125.9)	137.2	<0.0001 *	124.5	**<0.0001**
		4 weeks	**104.4**	(85.1, 123.7)	**196.3**	(145.8, 246.9)	**110.3**	(98.7, 130.8)	108.1	0.0001 *	96.8	0.0005 *
	**15-HEPE** **[pg/mL]**	Baseline	**33.9**	(21.9, 45.9)	**27.6**	(22.3, 32.9)	**31.3**	(21.9, 40.7)				
		2 days	**30.3**	(24.1, 36.5)	**49.6**	(38.1, 61.0)	**41.2**	(27.8, 54.7)	25.6	0.0002 *	12.1	0.1351 *
		4 weeks	**33.5**	(26.6, 40.3)	**46.4**	(31.0, 61.7)	**37.2**	(27.4, 47.0)	19.2	0.0924 *	12.9	0.3470 *
**DHA metabolites**	**4-HDHA** **[pg/mL]**	Baseline	**55.7**	(45.3, 66.2)	**53.5**	(44.7, 62.4)	**50.1**	(40.5, 59.7)				
		2 days	**51.3**	(43.5, 59.1)	**76.3**	(64.8, 87.8)	**55.4**	(44.1, 66.8)	27.2	0.0004 *	17.4	**0.0048**
		4 weeks	**53.5**	(45.5, 61.6)	**74.3**	(62.9, 85.7)	**60.8**	(50.3, 71.2)	22.9	**0.0014**	10.1	**0.1127**
	**10-HDHA** **[pg/mL]**	Baseline	**28.0**	(22.5, 33.5)	**31.6**	(25.6, 37.5)	**33.9**	(21.2, 46.6)				
		2 days	**30.0**	(24.1, 35.9)	**43.6**	(35.4, 51.9)	**37.7**	(23.5, 51.9)	10.1	0.0319 *	8.3	**0.0449**
		4 weeks	**28.4**	(23.6, 33.3)	**48.7**	(38.4, 59.1)	**36.1**	(28.7, 43.5)	16.8	0.0096 *	15.0	0.0516 *
	**17-HDHA** **[pg/mL]**	Baseline	**80.3**	(62.6, 98.0)	**89.5**	(71.2, 107.9)	**122.3**	(68.8, 175.8)				
		2 days	**86.5**	(70.8, 102.1)	**146.3**	(109.9, 182.6)	**125.6**	(61.2, 190.0)	50.7	0.0144 *	53.5	0.0246 *
		4 weeks	**107.1**	(82.3, 132.0)	**100.4**	(75.6, 125.2)	**125.3**	(87.0, 163.7)	−15.9	0.2770 *	7.9	0.9913 *

* Wilcoxon rank sum test; without *: ^§^ ANCOVA, ^#^ unpaired t-test; ** 15-HETE Fish oil n = 24 (outlier excluded).

**Table 3:** Total cholesterol, LDL-cholesterol, HDL-cholesterol and triglyceride concentrations at baseline, after 2 days and 4 weeks of supplementation presented by study groups. Mean ± 95% CI. Differences in mean changes between SynΩ3 and placebo as well as SynΩ3 and fish oil are shown.

Lipid Status	Assessment Points	Placebo (n = 24) Mean (95% CI)		SynΩ3 (n = 23) Mean (95% CI)		Fish Oil (n = 25) Mean (95% CI)		Difference in Mean Change SynΩ3 vs. Placebo	*p*-Value ^§^	Difference in Mean Change SynΩ3 vs. Fish Oil	*p*-Value ^#^
**Total cholesterol** **[mg/dL]**	**Baseline**	**210.3**	(192.5, 228.0)	**228.7**	(211.8, 245.7)	**224.4**	(207.7, 241.2)				
	4 weeks	**210.3**	(193.2, 227.3)	**224.4**	(205.9, 243.0)	**219.8**	(203.2, 236.4)	−4.3	**0.6624**	0.3	**0.9414**
**LDL** **[mg/dL]**	Baseline	**137.0**	(118.7, 155.3)	**156.1**	(140.6, 171.7)	**149.7**	(133.4, 166.1)				
	4 weeks	**136.9**	(118.7, 155.1)	**157.8**	(140.3, 175.3)	**148.6**	(131.9, 165.3)	1.8	**0.6233**	2.8	**0.5569**
**HDL** **[mg/dL]**	Baseline	**60.4**	(54.2, 66.6)	**58.5**	(52.8, 64.2)	**60.9**	(55.3, 66.6)				
	4 weeks	**63.5**	(56.4, 70.7)	**57.9**	(51.5, 64.3)	**61.7**	(55.5, 68.0)	−3.8	**0.0331**	−1.4	**0.3517**
**Triglycerides** **[mg/dL]**	Baseline	**105.3**	(74.9, 135.6)	**114.3**	(97.2, 131.3)	**104.2**	(82.2, 126.3)				
	4 weeks	**113.1**	(82.3, 144.0)	**102.0**	(81.9, 122.2)	**92.2**	(74.8, 109.7)	−20.1	0.1034*	−0.3	0.4387*

* Wilcoxon rank sum test; without *: ^§^ ANCOVA, ^#^ unpaired t-test.

## Data Availability

The data presented in this study are available on request from the corresponding author. The data are not publicly available due to patenting processes.

## Supplementary Materials

The following supporting information can be downloaded at: https://www.mdpi.com/article/10.3390/nu16091354/s1, Table S1: Total and free EPA and DHA concentrations at baseline, after 2 days and 4 weeks of supplementation presented by study groups; Table S2: TNF and hsCRP concentrations at baseline, after 2 days and 4 weeks of supplementation presented by study groups.
