# Supplementary material for: Synbiotic Bacillus megaterium DSM 32963 and n-3 PUFA Salt Composition Elevates Pro-Resolving Lipid Mediator Levels in Healthy Subjects: A Randomized Controlled Study
Source: Nutrients. 2024 Apr 30;16(9):1354. doi: 10.3390/nu16091354 (PMC11085393; doi:10.3390/nu16091354)
Supplement: Supplementary file 1 [file nutrients-16-01354-s001.zip › Supplementary Table S1.pdf]

Supplementary Table S1: Total and free EPA and DHA concentrations in plasma at baseline, after 2 days and 4 weeks of supplementation presented by study groups. Mean ± 95% CI. Differences in mean changes between SynΩ3 and placebo as well as SynΩ3 and Fish oil are shown.

***n*-3 PUFA**	**Assessment points**	**Placebo (n=24) Mean (95% CI)**		**SynΩ3 (n=23) Mean (95% CI)**		**Fish oil (n=25) Mean (95% CI)**		Difference in mean change SynΩ3 vs. Placebo	p-value§	Difference in mean change SynΩ3 vs. Fish oil	p-value#
**total EPA [µg/mL]**	Baseline	**21.9**	(18.5, 25.4)	**25.0**	(21.3, 28.7)	**24.3**	(20.4, 28.3)				
	2 days	**21.5**	(18.2, 24.8)	**26.3**	(22.3, 30.2)	**30.1**	(25.8, 34.3)	1.7	**0.0445**	-4.5	**<0.0001**
	4 weeks	**26.4**	(20.7, 32.1)	**30.3**	(25.4, 35.2)	**37.9**	(33.6, 42.3)	0.8	**0.4187***	-8.4	**0.0055**
**total DHA [µg/mL]**	Baseline	**45.3**	(40.2, 50.5)	**51.8**	(44.4, 59.2)	**45.7**	(40.9, 50.5)				
	2 days	**45.9**	(40.7, 51.1)	**52.4**	(45.3, 59.5)	**47.4**	(43.0, 51.7)	0.0	**0.7073**	-1.1	**0.4535**
	4 weeks	**48.0**	(41.1, 54.9)	**54.9**	(47.6, 62.1)	**55.6**	(50.0, 61.2)	0.5	**0.8315***	-6.8	**0.0173**
**free EPA [ng/mL]**	Baseline	**621.7**	(452.7, 790.7)	**593.3**	(479.4, 707.2)	**646.6**	(546.9, 746.3)				
	2 days	**640.9**	(481.6, 800.2)	**748.9**	(609.3, 888.5)	**742.7**	(633.6, 851.8)	136.4	**0.0698**	59.5	**0.3968**
	4 weeks	**670.6**	(547.6, 793.7)	**810.3**	(661.3, 959.2)	**844.4**	(703.4, 985.3)	168.0	**0.0676**	19.2	**0.7988**
**free DHA [ng/mL]**	Baseline	**1307.0**	(1083, 1531)	**1384.0**	(1177, 1591)	**1315.0**	(1144, 1486)				
	2 days	**1227.0**	(1033, 1420)	**1406.0**	(1178, 1633)	**1377.0**	(1169, 1584)	102.0	**0.2069**	-40.0	**0.6535**
	4 weeks	**1374.0**	(1149, 1598)	**1452.0**	(1245, 1660)	**1499.0**	(1206, 1791)	1.0	**0.7530**	-116.0	**0.4390***

* Wilcoxon rank sum test; without *: §ANCOVA, #unpaired t-test
