# Supplementary material for: Synbiotic Bacillus megaterium DSM 32963 and n-3 PUFA Salt Composition Elevates Pro-Resolving Lipid Mediator Levels in Healthy Subjects: A Randomized Controlled Study
Source: Nutrients. 2024 Apr 30;16(9):1354. doi: 10.3390/nu16091354 (PMC11085393; doi:10.3390/nu16091354)
Supplement: Supplementary file 1 [file nutrients-16-01354-s001.zip › Supplementary Table S2.pdf]

Supplementary Table S2: TNF and hsCRP concentrations at baseline, after 2 days and 4 weeks of supplementation presented by study groups. Mean ± 95% CI. Differences in mean changes between SynΩ3 and placebo as well as SynΩ3 and Fish oil are shown.

**Inflammatory marker**	**Assessment points**	**Placebo (n=24) Mean (95% CI)**		**SynΩ3 (n=23) Mean (95% CI)**		**Fish oil (n=25) Mean (95% CI)**		Difference in mean change SynΩ3 vs. Placebo	p-value§	Difference in mean change SynΩ3 vs. Fish oil	p-value#
**TNF-α [pg/mL]**	Baseline	**6.05**	(5.42, 6.69)	**6.14**	(5.41, 6.86)	**6.17**	(5.66, 6.68)				
	2 days	**6.09**	(5.40, 6.79)	**5.95**	(5.33, 6.58)	**6.04**	(5.57, 6.52)	-0.22	**0.3600**	-0.06	**0.7652**
	4 weeks	**6.03**	(5.26, 6.81)	**6.23**	(5.63, 6.83)	**6.09**	(5.62, 6.56)	0.12	**0.5793***	0.18	**0.4541**
**hsCRP [mg/L]**	Baseline	**2.46**	(1.09, 3.83)	**1.56**	(0.90, 2.22)	**1.70**	(0.92, 2.47)				
	2 days	**2.09**	(1.17, 3.00)	**1.71**	(0.79, 2.64)	**1.46**	(0.87, 2.04)	0.53	**0.2592***	0.39	**0.7721***
	4 weeks	**3.46**	(1.44, 5.48)	**1.93**	(0.99, 2.88)	**1.40**	(0.87, 1.93)	-0.63	**0.7333***	0.67	**0.0862***

* Wilcoxon rank sum test; without *: §ANCOVA, #unpaired t-test
